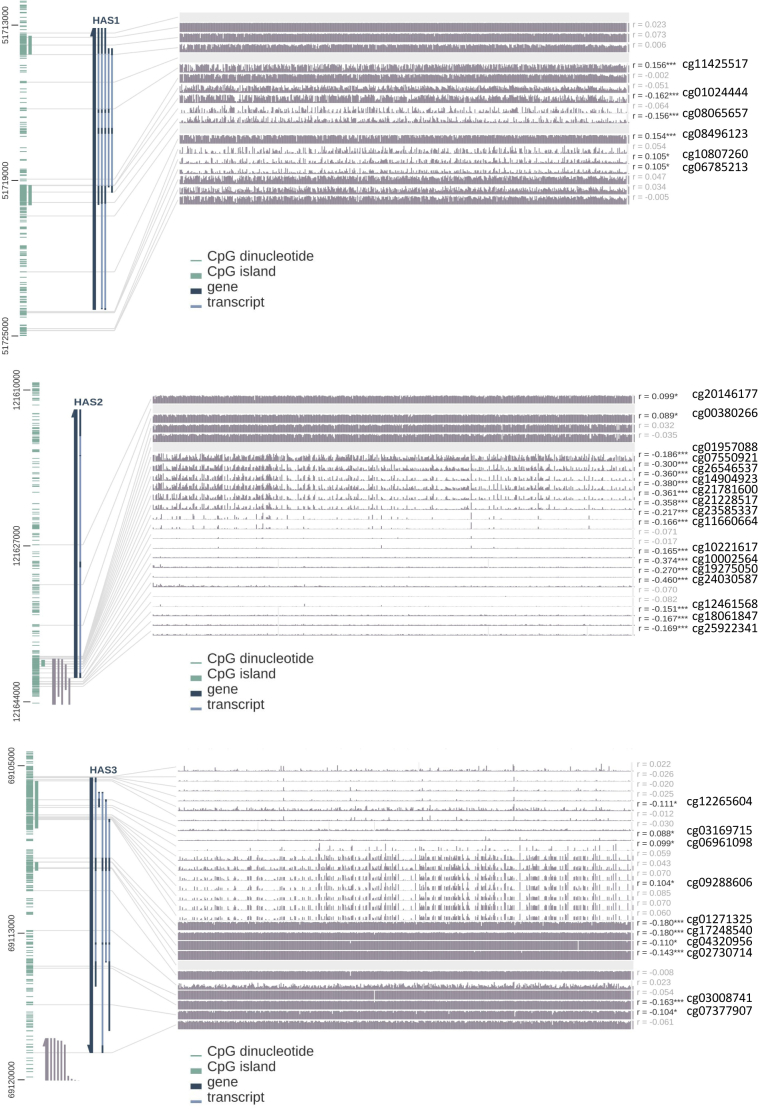# Pan-cancer analysis reveals the potential of hyaluronate synthase as therapeutic targets in human tumors

**DOI:** 10.1016/j.heliyon.2023.e19112

**Published:** 2023-08-12

**Authors:** Xunxia Bao, Juan Ran, Chuifang Kong, Zunxi Wan, Juling Wang, Tengfei Yu, Shengming Ruan, Wenjing Ding, Leiming Xia, Daoxiang Zhang

**Affiliations:** a School of Life Sciences, Anhui Medical University, Hefei, 230032, China; b School of Life Sciences, Northeast Normal University, Changchun, 130024, China; c Department of Hematology, The First Affiliated Hospital of Anhui Medical University, Hefei, 230022, China

**Keywords:** Hyaluronate synthase, Pan-cancer, Tumor microenvironment, Extracellular matrix, Prognosis, Immune, Glioblastoma multiforme

## Abstract

Hyaluronic acid (HA) is a crucial component of the extracellular matrix, and its level of accumulation is related to the progression of various malignant tumors. In this study, a pan-cancer analysis of the three enzymes called hyaluronan synthases (HAS1, HAS2, and HAS3) that produce HA was performed. The study comprehensively describes the characteristics of HAS1, HAS2, and HAS3 in cancers using public databases and tools, to identify the potential biological pathways involved at the molecular, protein, cellular, and clinical sample levels. The analysis showed that dysregulation of the three genes often occurs in cancer, contributing to cancer progression, metastasis, and prognosis. Overexpression of HAS2 promotes secretion of HA in GBM and enhances cell proliferation and migration. The common and specific functions of HAS in certain diseases have important research implications for the treatment and prognosis of tumors.

## 1 Introduction

The chemoresistance of tumor cells is affected by the tumor microenvironment (TME) [1] composed of extracellular matrix (ECM), fibroblasts, tumor vasculature system, and immune cells, all of which participate in regulating tumor development. The accumulation level of hyaluronic acid (HA), an important component of the ECM, has been found to be closely related to the accelerated proliferation, invasion, and metastasis of tumor cells in various malignant tumors [2]. The abnormal increase of HA in the matrix can compress the microvasculature system inside the tumor, lead to increased stromal fluid pressure, inhibit drug delivery ability, and thus confer drug resistance to tumor cells [3]. Since the metabolism of HA is crucial in regulating cancer signaling and cellular behavior, it is necessary to evaluate its relevance to cancer prognosis and explore the related molecular mechanisms.

HA is synthesized and released into the ECM by three types of transmembrane isoenzymes, namely hyaluronan synthases (HAS1, HAS2, and HAS3), which repeatedly add d-glucuronic acid and N-acetyl-d-glucosamine [4]. The different subtypes of HAS synthesize HA of different lengths. HAS1 and HAS2 synthesize high molecular weight polymers greater than 2000 kDa, while HAS3 produces low molecular weight polymers less than 200 kDa [5]. In addition to polymer length, the catalytic activity and regulatory roles of the three subtypes are not entirely identical. Extensive research has shown that HAS expression plays an important role in carcinogenesis [[6], [7], [8]].

Pan-cancer analysis is often used to initially explore the differential expression and clinical significance of genes of interest in various tumors. In addition, it enables the identification of key biological processes that contribute to the dysregulation of the TME [9]. In this study, we utilized various databases and tools including University of California Santa Cruz (UCSC), National Center for Biotechnology Information (NCBI), The Cancer Genome Atlas (TCGA), Genotype-Tissue Expression (GTEx), HomeloGene, COBALT, Human Protein Atlas (HPA), gene expression profiling interactive analysis (GEPIA2), UALCAN, Kaplan-Meier Plotter (KM Plotter), cBioPortal, Tumor immune estimation resource version 2.0 (TIMER2.0), Genomic Data Commons (GDC), SangerBox, Methylation and Expression Explorer (MEXPRESS), Simple Modular Architecture Research Tool (SMART), search tool for the retrieval of interacting genes/proteins (STRING), Metascape, and PhosphoSitePlus, to comprehensively analyze the correlation between the mRNA and protein levels of the genes HAS1, HAS2, HAS3 and their association with pan-cancer prognosis, mutation, methylation, phosphorylation, immune infiltration, and potential molecular mechanisms. In addition, we performed knockout (KO) and overexpression (OE) of HAS2 in glioblastoma multiforme (GBM) cells, and observed the effects of these alterations on the behavior of cancer cells. The objective of this study is to investigate the expression changes of HAS genes in different cancer types, explore their correlation with prognosis, and uncover potential underlying mechanisms. Furthermore, provide theoretical evidence for the potential use of HAS genes as prognostic, diagnostic, or therapeutic biomarkers in cancer.

To our knowledge, this is the first pan-cancer analysis focusing on the value of HAS in human tumors. Our findings demonstrate the important roles of different HAS types in specific diseases, and suggest that targeting their gene expression or encoded proteins, or designing therapeutic strategies in conjunction with their pathways and associated networks, may be helpful in inhibiting cancer progression.

## 2 Materials and methods

### 2.1 Overview of the gene location, protein structure, and evolutionary relationships of HAS

UCSC Genome Browser can quickly and accurately display the requested portion of the genome at any scale, and integrate a large number of aligned annotation tracks [10]. We entered different HAS types into the search box and selected to visualize the specific location of the genes on the chromosomes in the human species (GRCh38/hg38). Next, we used the “HomoloGene” module in NCBI to compare the conserved domain types and amino acid (aa) sizes of homologous genes across multiple species. In addition, we used the COBALT tool in NCBI to align protein sequences and generate a systematic evolutionary tree.

### 2.2 Expression levels of HAS in different tissues and multiple cancers

The HPA database [11], based on multi-omics methods, provides immunohistochemical images and protein data from different tissues, cell types, and subcellular organelles. By searching different types of HAS proteins in the HPA database, we show the distribution of these proteins in all major tissues and organs of the human body, as well as the expression of their encoding genes in immune cell types. The TIMER2.0 database [12] displays the expression differences of HAS between specific tumors or their subtypes and adjacent normal tissues in the TCGA database. For tumors that lacked sufficient normal tissue samples, we used the GEPIA2 tool [13] to obtain intergroup differences. The |Log2FC| cutoff was set to 1, the p-value cutoff was set to 0.01, and the tumor dataset was matched with the TCGA and GTEx normal tissue data for analysis, resulting in box plots representing intergroup differences. In addition, we calculated the expression differences of HAS in each tumor among different clinical stages samples (I, II, III, and IV). We used non-paired Student's *t*-Test to analyze the significance of differences between pairs, and performed analysis of variance (ANOVA) to test for differences among multiple groups of samples.

### 2.3 Survival curve

The “survival analysis” module of GEPIA2 tool was used to evaluate the survival contribution of HAS in various cancers. Cutoff-High (%) and Cutoff-Low (%) were both set to 50, the significance level was 0.05, and time unit was set as months. Survival maps were obtained for both overall survival (OS) and disease free survival (DFS). The KM Plotter [14] can assist researchers in conducting cancer survival analysis, and explore the expression of genes, miRNAs, and proteins related to disease survival rates. It collects cancer patient records from public gene expression databases such as GEO, European Genome-Phenome Archive (EGA), and TCGA, and provides KM survival curves. We used it to statistically analyze the correlation between the expression levels of three HAS genes and OS/DFS in 21 types of cancer, and the best cutoff was automatically selected.

### 2.4 Prognostic biomarker correlation analysis in cancer

We downloaded a pan-cancer dataset, TCGA Pan-Cancer (PANCAN, N = 10535, G = 60499), from the UCSC database after applying uniform normalization. HAS1, 2, and 3 gene expression data were extracted from each sample. Simple nucleotide variation data (Level 4) were obtained for all TCGA samples processed by MuTect2 software [15] from the GDC database. The tumor mutational burden (TMB) was calculated for each tumor using the ‘tmb’ function of the ‘maftools (version 2.8.05)' R package. The microsatellite instability (MSI) score of tumors was obtained from the study by Russell Bonneville et al. [16]. TMB or MSI from each sample and expression data from three genes were integrated. Each expression value was transformed by log2 (x+0.001) and cancers with fewer than 3 samples in a single type were excluded. Finally, we obtained the expression data of 37 cancers and generated a histogram using Sangerbox [17].

### 2.5 Genetic alteration analysis

cBioPortal [18] provides data for multiple cancer types, including gene mutations, protein expression, copy number alteration (CNA), and clinical data, to help users gain a deep understanding of the bioinformatics features and treatment strategies of tumors. We selected the “TCGA Pan Cancer Atlas Studies” option in the cBioPortal database and entered “HAS1″, “HAS2″, and “HAS3″ to query their mutation frequency, mutation type, CNA results, and mutation sites in the tumor.

### 2.6 Correlation between HAS and DNA methyltransferase 1 (DNMT1) and the analysis of methylation sites

We used the “correlation analysis” tool in GEPIA2 to obtain significant correlations between HAS and DNMT1 in certain types of tumors. Next, we analyzed and visualized the relationship between DNA methylation profiles and HAS gene expression using MEXPRESS [19,20].

### 2.7 Overview of HAS protein phosphorylation, acetylation, and ubiquitination

The PhosphoSitePlus database [21] is a publicly available database that specializes in collecting and maintaining information on protein phosphorylation modifications. The database collects information from scientific literature, large-scale datasets, and manual annotations to provide relevant information for the study of post-translational modification (PTM), including phosphorylation, ubiquitination, and acetylation. We used this database to query the known sites of HAS phosphorylation, ubiquitination, and acetylation as documented in literature.

### 2.8 Immune infiltration

TIMER 2.0 utilizes seven immune infiltration algorithms (TIMER, CIBERSORT, CIBERSORT-ABS, QUANTISEQ, XCELL, MCPCOUNTER and EPIC) to evaluate the degree of immune cell infiltration and the expression levels of key genes affecting tumor development in tumor tissues. We used TIMER 2.0 to summarize the correlation between three HAS genes and immune cell infiltration in TCGA tumors.

### 2.9 Pathway enrichment and co-expression network

The STRING database [22] was used to predict interactions among proteins, genes and complexes, and to annotate functions such as biological pathways, regulatory networks, and biological processes. HAS1, HAS2, and HAS3 were separately inputted under the classification of “*Homo sapiens*”, with a minimum required interaction score set to low confidence (0.150), and active interaction sources were set as experimental data. The top 20 reliable interacting proteins were finally obtained. Then, GEPIA2 was used to predict the top 100 genes with similar expression patterns in different cancer types and normal tissues for the three genes. The intersecting genes of the top 100 genes for the three genes were obtained using http://bioinformatics.psb.ugent.be/webtools/Venn/, and the correlation and p-values were displayed. Next, we uploaded the list of 120 genes (each HAS gene resulted in 20 interacting proteins and 100 similar proteins) into the metascape tool for analysis, and outputted results including enrichment analysis charts and data tables. Kyoto Encyclopedia of Genes and Genomes (KEGG), Gene Ontology Biological Process (GOBP), Gene Ontology Cellular Component (GOCC), and Gene Ontology Molecular Function (GOMF) bubble charts were plotted. Finally, the functionally enriched genes were identified for the top 5 GOBP, GOCC, and GOMF categories.

### 2.10 Cell lines

The human normal astrocyte cell line (HA1800) and GBM cell lines (U87, U118, U138, and U251) were purchased from BeNa Culture Collection (Beijing, China). Western blot was used to detect the expression level of HAS2 in the above-mentioned cell lines. U138 cell lines with HAS2-OE and HAS2-KO were separately constructed for subsequent experiments.

### 2.11 Particle exclusion assay

Cells were seeded at a density of 2000 cells/well in a 6-well plate and incubated in a 37 °C, 5% CO2 incubator for 24 h. After adding 500 μl sheep red blood cells and mixing thoroughly, the plate was further incubated for 1 h at 37 °C, 5% CO2. The non-adherent sheep red blood cells and medium mixture were discarded, and 1 ml of 10% fetal bovine serum (FBS) culture medium was added along the wall. The secretion of HA by cells appeared as clear spots under a microscope. The area of the clear spots was calculated using Image J software.

### 2.12 Soft agar assay

Agar medium (0.6%) was added to the bottom of a 24-well plate and 0.3% agar medium containing 1000 cells was added to the top layer. Incubate at 37 °C, 5% CO2 for 1 week, then photograph and count the number of clones under a microscope.

### 2.13 Wound healing assay

The surface of a 6-well plate filled with cells was scratched using a 10 μl pipette tip. Discard the culture medium, wash twice with PBS, and add DMEM. Take photographs at 0 and 48 h. Measure the migration rate of cells during this period. The migration rate is calculated as (width of wound at 0 h - width of wound at 48 h)/width of wound at 0 h.

### 2.14 Statistical analysis

The data obtained from the experiment were analyzed and presented using GraphPad Prism (version 8.0, San Diego, USA) to draw histograms. The experimental results were obtained from three replications. The difference between two groups was analyzed using Student's *t*-test. A p-value less than 0.05 was considered statistically significant (*P < 0.05, **P < 0.01, ***P < 0.001, ****P < 0.0001).

## 3 Results

### 3.1 Structural features and phylogenetic tree of HAS1, 2, and 3 in different species

GRCh38/hg38 is the current widely used version of the human genome. Fig. S1a shows that the three genes are not located in the same position on the chromosome. HAS1 is located on human chromosome 19q13.41 (50900001–53100000 interval, mRNA NM_001297436.2, protein NP_001284365.1), HAS2 is located on human chromosome 8q24.13 (118300001–121500000 interval, mRNA NM_005328.3, protein NP_005319.1), and HAS3 is located on human chromosome 16q22.1 (88700001–90338345 interval, mRNA NM_001199280.2, protein NP_001186209.1). The structural features of the three HAS proteins are conserved among different species, such as *Homo sapiens* (*H. sapiens*), *Mus musculus* (*M. musculus*), and *Danio rerio* (*D. rerio*), usually containing the glycosyltransferase family A (GT-A) domain (Fig. S1b). To better understand the evolutionary relationships of the three HAS proteins among various species, a phylogenetic tree (Fig. S2) was constructed.

### 3.2 HAS1, 2, and 3 show similar distribution patterns in human tissues but have RNA blood cell type specificity

The distribution patterns of the three HAS genes were then examined across 55 different human tissue types. Based on data from the Human Protein Atlas (HPA) and Genotype-Tissue Expression (GTEx) transcriptome databases, these tissue types were grouped together based on their shared functional characteristics. HAS1 and HAS2 exhibited the highest expression levels in adipose tissue, whereas HAS3 displayed the highest expression level in the esophagus. Additionally, HAS1 showed relatively high distribution in the ovary, while HAS2 and HAS3 exhibited relatively high distribution in the urinary bladder (Fig. S3a).

Furthermore, the expression patterns of the HAS genes were investigated in 29 blood cell types, as well as in total peripheral blood mononuclear cells (PBMCs). As depicted in Fig. S3b, HAS1 and HAS2 demonstrated significant RNA blood cell type specificity, primarily found in granulocytes. While HAS3 was distributed in T-cells, monocytes, granulocytes, B-cells, dendritic cells, NK-cells, total PBMCs, and progenitors.

### 3.3 Expression levels of HAS1, 2, and 3 differ in different types of tumors

To obtain a more profound understanding of the involvement of HAS genes in tumor biology, we conducted a comprehensive analysis by examining the expression profiles of HAS genes across diverse cancer types and their corresponding adjacent normal tissues relied upon the TCGA (The Cancer Genome Atlas) database (Fig. 1a). In cases where certain tumor types lacked or had insufficient normal control samples, TCGA normal and GTEx data were utilized as substitutes for controls. These results are visually depicted in Fig. 1b, c, d (where significant differences were observed) and Figs. S4a, b, c (where significant differences were not observed).

**Fig. 1:**
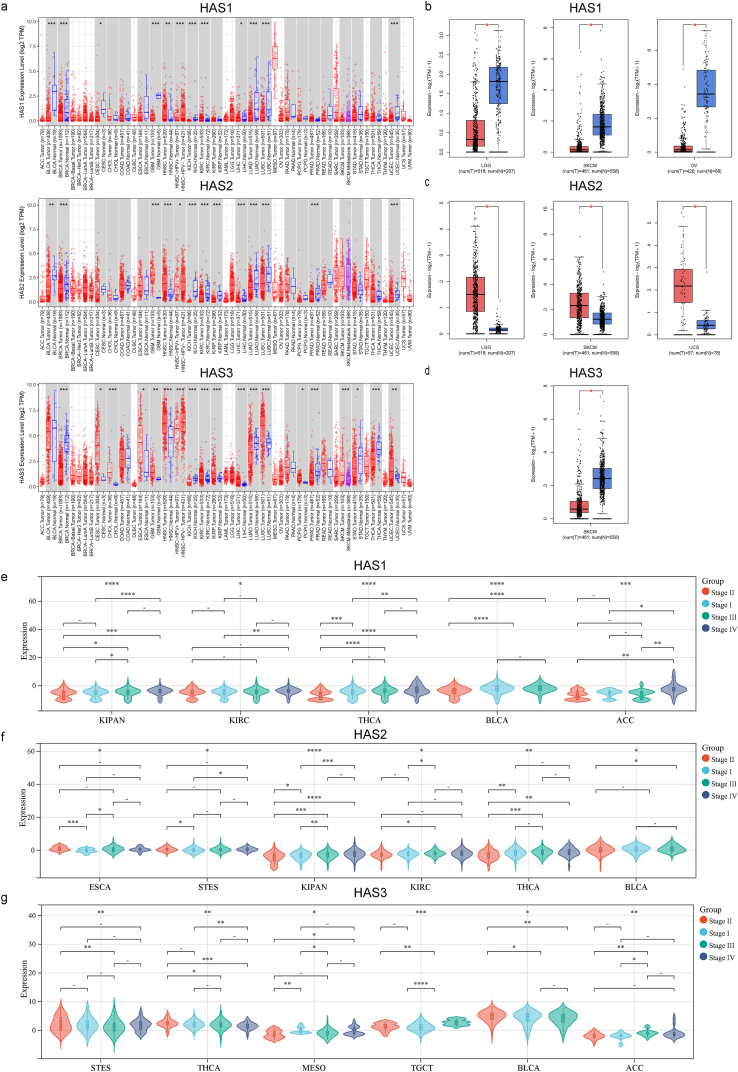
Expression level of HAS1,2, and 3 in pan-cancer and pathological stages. (a) The TIMER2.0 database was used to display the differential expression of three types of HAS between specific tumors or their subtypes and adjacent normal tissues. When there are data for normal controls in tumors, they are shown together in a gray area. The red box reflects the gene expression in tumors, and the blue box reflects the gene expression in normal tissues; (b) significant downregulation of HAS1 in tumor tissues of LGG, OV, SKCM; (c) significant upregulation of HAS2 in tumor tissues of LGG, SKCM, and UCS; (d) significant downregulation of HAS3 in tumor tissues of SKCM; tumor types associated with the expression levels of HAS1 (e), HAS2 (f), and HAS3 (g) in different pathological stages (Stage I, II, III, and IV). *P < 0.05, **P < 0.01, ***P < 0.001, ****P < 0.0001.

Based on the aforementioned findings, we observed distinct expression patterns of HAS1, HAS2, and HAS3 across various cancer types. HAS1 exhibited significant downregulation in bladder urothelial carcinoma (BLCA), breast invasive carcinoma (BRCA), cervical squamous cell carcinoma and endocervical adenocarcinoma (CESC), glioblastoma multiforme (GBM), kidney chromophobe (KICH), liver hepatocellular carcinoma (LIHC), lung adenocarcinoma (LUAD), lung squamous cell carcinoma (LUSC), uterine corpus endometrial carcinoma (UCEC), brain lower grade glioma (LGG), ovarian serous cystadenocarcinoma (OV), and skin cutaneous melanoma (SKCM). Conversely, HAS1 was significantly upregulated in head and neck squamous cell carcinoma (HNSC) and kidney renal clear cell carcinoma (KIRC). Similarly, HAS2 exhibited significant downregulation in BLCA, BRCA, KICH, KIRC, kidney renal papillary cell carcinoma (KIRP), LIHC, LUAD, LUSC, prostate adenocarcinoma (PRAD), and UCEC tumors. In contrast, it was significantly upregulated in GBM, HNSC, LGG, SKCM, and uterine carcinosarcoma (UCS) tumors. As for HAS3, it showed significant downregulation in BRCA, KICH, LUAD, PRAD, stomach adenocarcinoma (STAD), thyroid carcinoma (THCA), and SKCM tumors. Conversely, HAS3 exhibited significant upregulation in CESC, cholangiocarcinoma (CHOL), esophageal carcinoma (ESCA), HNSC, KIRC, KIRP, LIHC, LUSC, pheochromocytoma and paraganglioma (PCPG), and UCEC tumors. Notably, breast invasive carcinoma (BRCA), kidney chromophobe (KICH), and lung adenocarcinoma (LUAD) showed significant downregulation of all three HAS genes, while head and neck squamous cell carcinoma (HNSC) exhibited significant upregulation of all three genes. Furthermore, the expression levels of all three genes were found to be correlated with cancer pathological staging. In particular, thyroid carcinoma (THCA) and BLCA displayed significant correlations with all three genes, as depicted in Fig. 1e, f, g. Conversely, certain cancer types, such as lymphoid neoplasm diffuse large B-cell lymphoma (DLBC), acute myeloid leukemia (LAML), sarcoma (SARC), and thyroid carcinoma (THYM), showed no significant changes in the expression of all three genes. These associations are illustrated in Figs. S4d, e, f.

### 3.4 Survival analysis indicates that the expression of three HAS genes is associated with different prognosis in various tumor cases

To assess the relative contributions of the three genes to patient survival across different cancer types, we employed the GEPIA2 tool and KM Plotter database to conduct separate prognostic analyses. In the TCGA project, high expression of HAS1 was associated with poor overall survival (OS) in adrenocortical carcinoma (ACC), KIRP, and poor OS and DFS in GBM, KIRC (Fig. 2a). High expression of HAS2 was associated with poor OS in LUSC, MESO, STAD, and poor DFS in OV, as well as poor OS and DFS in KIRP, LGG, SARC (Fig. 2b). High expression of HAS3 was associated with poor OS in MESO, but indicated better survival outcomes in DFS of LUAD and PRAD (Fig. 2c). In addition, we integrated 7462 cancer samples from the KM Plotter database for OS analysis and 4420 cancer samples for DFS analysis, and compiled Table 1, where items with p < 0.05 were marked in red.

**Fig. 2:**
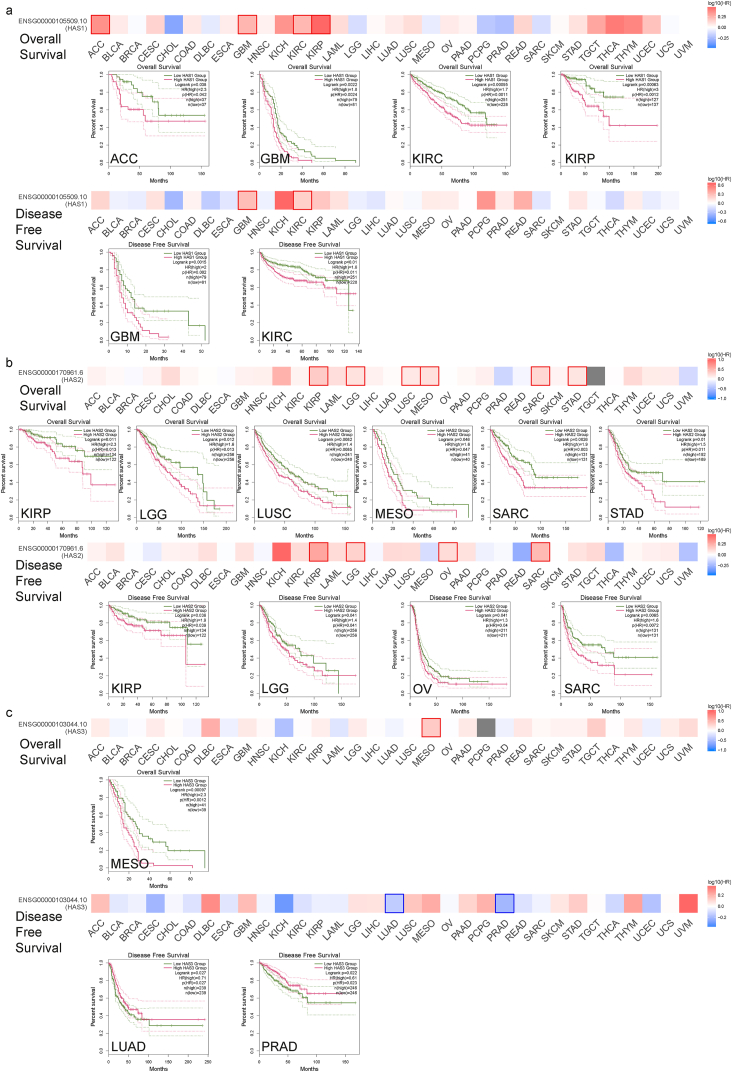
Survival maps and KM curves showed tumor types associated with HAS1 (a), HAS2 (b), and HAS3 (c) of overall survival and disease free survival.

**Table 1:** Prognostic values of HAS1, 2, 3 in pan-cancer using the Kaplan-Meier database.

	HAS1				HAS2				HAS3			
	Overall Survival (OS)		Disease Free Survival (DFS)		Overall Survival (OS)		Disease Free Survival (DFS)		Overall Survival (OS)		Disease Free Survival (DFS)	
Cancer type	HR	Logrank P	HR	Logrank P	HR	Logrank P	HR	Logrank P	HR	Logrank P	HR	Logrank P
Bladder Carcinoma	1.38 (1–1.91)	0.051	0.28 (0.13–0.58)	0.00024	1.61 (1.18–2.19)	0.0026	0.48 (0.22–1.01)	0.047	0.64 (0.48–0.87)	0.0038	0.39 (0.19–0.81)	0.0091
Breast cancer	0.75 (0.53–1.05)	0.087	0.6 (0.39–0.93)	0.021	0.76 (0.55–1.05)	0.098	0.76 (0.49–1.2)	0.24	0.69 (0.49–0.97)	0.034	0.72 (0.43–1.18)	0.19
Cervical squamous cell carcinoma	1.52 (0.86–2.68)	0.15	5.4 (1.28–22.86)	0.01	2.15 (1.15–4.01)	0.013	0.65 (0.29–1.45)	0.29	1,47 (0.9-2.4)	0.12	0.49 (0.22–1.07)	0.068
Esophageal Adenocarcinoma	1.86 (0.9–3.84)	0.091	10.45 (1.08–100.87)	0.012	2.59 (1.21–5.51)	0.011	7.27 (0.74–71.67)	0.049	0.71 (0.34–1.47)	0.35	0.14 (0.01–1.42)	0.057
Esophageal Squamous Cell Carcinoma	0.5 (0.21–1.16)	0.098	0.65 (0.24–1.75)	0.39	0.71 (0.3–1.71)	0.44	1.93 (0.63–5.93)	0.24	1.69 (0.72–3.97)	0.23	0.28 (0.08–0.97)	0.032
Head-neck squamous cell carcinoma	1.3 (0.98–1.73)	0.066	0.45 (0.21–0.94)	0.029	1.35 (1.04–1.77)	0.026	1.55 (0.71–3.36)	0.26	1.5 (1.11–2.04)	0.0087	0.57 (0.26–1.22)	0.14
Kidney renal clear cell carcinoma	2.28 (1.61–3.24)	2.2E-06	0.24 (0.05–1.07)	0.042	1.55 (1.14–2.12)	0.0048	0.47 (0.13–1.69)	0.24	1.39 (1.01–1.92)	0.042	0.23 (0.07–0.75)	0.0078
Kidney renal papillary cell carcinoma	3.57 (1.97–6.45)	7E-06	3.62 (1.36–9.66)	0.0061	2.84 (1.52–5.31)	0.00063	2.16 (1.01–4.6)	0.041	2.07 (1.14–3.75)	0.015	1.86 (0.85–4.05)	0.12
Liver hepatocellular carcinoma	1.51 (1.02–2.23)	0.038	0.66 (0.48–0.93)	0.016	1.47 (0.97–2.24)	0.069	0.79 (0.56–1.12)	0.18	1.55 (1.08–2.24)	0.017	1.42 (1.02–1.97)	0.037
Lung adenocarcinoma	1.27 (0.95–1.71)	0.11	0.72 (0.48–1.1)	0.13	1.22 (0.87–1.69)	0.25	1.55 (0.93–2.6)	0.09	0.75 (0.53–1.05)	0.087	0.56 (0.36–0.85)	0.0062
Lung squamous cell carcinoma	1.61 (1.22–2.11)	0.00056	1.74 (0.97–3.12)	0.061	1.51 (1.14–1.99)	0.0035	0.61 (0.33–1.13)	0.12	0.8 (0.59–1.07)	0.13	1.57 (0.95–2.59)	0.077
Ovarian cancer	0.74 (0.55–0.99)	0.045	0.68 (0.45–1.03)	0.066	1.15 (0.88–1.5)	0.32	1.59 (1.11–2.27)	0.011	0.84 (0.62–1.13)	0.25	0.8 (0.56–1.15)	0.23
Pancreatic ductal adenocarcinoma	0.76 (0.46–1.25)	0.28	0.7 (0.31–1.6)	0.4	1.8 (1.16–2.79)	0.0082	11.76 (1.58–87.66)	0.0023	2.21 (1.39–3.52)	0.00061	3.91 (1.14–13.41)	0.02
Pheochromocytoma and Paraganglioma	0.27 (0.04–1.61)	0.12	0.23 (0.03–1.66)	0.11	0.39 (0.08–1.94)	0.23	0 (0-lnf)	0.049	10.86 (1.26–93.29)	0.0066	0.3 (0.04–2.17)	0.21
Rectum adenocarcinoma	0.72 (0.31–1.66)	0.44	3.2 (037–28.05)	0.27	0.48 (0.21–1.1)	0.076	0.28 (0.04–2.09)	0.19	1.51 (0.57–4)	0.41	4.57 (0.83–5.09)	0.055
Sarcoma	0.61 (0.4–0.95)	0.028	0.41 (0.22–0.75)	0.0028	2.46 (1.64–3.69)	7.1E-06	1.63 (0.97–2.74)	0.063	1.2 (0.81–1.79)	0.36	1.33 (0.79–2.26)	0.28
Stomach adenocarcinoma	1.76 (1.26–2.45)	0.00073	1.98 (0.87–4.5)	0.098	1.9 (1.3–2.77)	0.00069	1.42 (0.74–2.72)	0.29	1.5 (1.01–2.22)	0.043	0.74 (0.39–1.41)	0.35
Testicular Germ Cell Tumor	0 (0-lnf)	0.12	2.09 (0.98–4.46)	0.051	65118565988 (0-lnf)	0.037	1.93 (0.9–4.15)	0.086	4.88 (0.5–47.58)	0.13	0.78 (0.35–1.7)	0.52
Thymoma	4.06 (0.84–19.63)	0.06	NA	NA	3.08 (0.77–12.31)	0.094	NA	NA	0.35 (0.09–1.33)	0.11	NA	NA
Thyroid carcinoma	4.61 (1.59–13.36)	0.0021	0.5 (0.23–1.09)	0.075	0.46 (0.17–1.26)	0.12	0.49 (0.23–1.07)	0.067	2.44 (0.91–6.57)	0.068	0.47 (0.18–1.26)	0.13
Uterine corpus endometrial carcinoma	2.6 (1.46–4.55)	0.00052	1.91 (1.14–3.22)	0.013	1.5 (0.99–2.28)	0.054	1.32 (0.78–2.24)	0.3	0.71 (0.46–1.1)	0.12	0.6 (0.36–1.01)	0.054

### 3.5 Gene alteration analysis of HAS1, 2, and 3

**Fig. 3:**
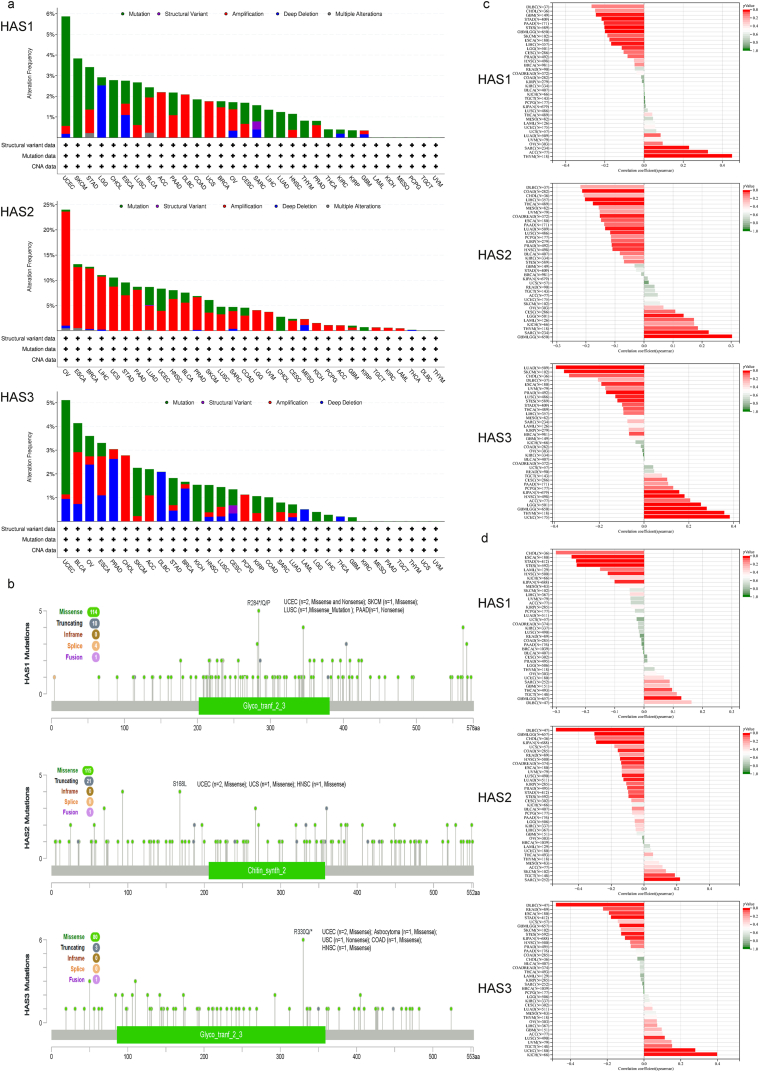
Gene alteration and TMB/MSI correlation analysis of HAS1, 2, and 3. (a) Alteration frequency and type of HAS; (b) mutation site of HAS; Spearman correlation of HAS expression with TMB (c) and MSI (d) in TCGA tumors.

We observed three types of gene mutations in the pan-cancer samples of the TCGA cohort. As shown in Fig. 3a, HAS1 and HAS3 exhibited the highest frequency of “mutation” style changes (>5%) in UCEC. HAS2, on the other hand, had amplification changes in most cancers, with OV having the highest change frequency (>20%). Furthermore, we integrated the types of gene mutations, locations, and number of cases, with missense mutations being the most frequent (Fig. 3b). In 2 cases of UCEC, 1 case of SKCM, 1 case of LUSC, and 1 case of pancreatic adenocarcinoma (PAAD), a nonsense or missense mutation was detected in the glycogenin-2-3 domain of HAS1 at amino acid position 284, where arginine (R) was replaced by a stop codon (*), glutamine (Q), or proline (P), resulting in a premature termination or alteration of the amino acid sequence. In 2 cases of UCEC, 1 case of UCS, and 1 case of HNSC, a missense mutation was detected in the chitin_synth_2 domain of HAS2 at amino acid position 168, where serine (S) was replaced by leucine (L). In 2 cases of UCEC, 1 case of astrocytoma, 1 case of colon adenocarcinoma (COAD), and 1 case of HNSC, a missense or nonsense mutation was detected in the glycogenin-2-3 domain of HAS3 at amino acid position 330, where arginine (R) was replaced by Q or *.

Tumor mutational burden (TMB) and microsatellite instability (MSI) are both indicators used to evaluate the prognosis and treatment response of cancer immunotherapy patients. TMB is an indicator for assessing the mutation load in tumors, while MSI is an indicator for evaluating the tumor DNA mismatch repair ability. Patients with elevated TMB and MSI typically have a better response to immune checkpoint inhibitors. The Spearman correlation between HAS expression and TMB (Fig. 3c) or MSI (Fig. 3d) was analyzed in all TCGA tumors. HAS1 was observed to be significantly positively correlated with TMB in three types of tumors, namely SARC, THYM, and ACC; and significantly negatively correlated with TMB in eight types of tumors, namely GBM, glioma (GBMLGG), LGG, ESCA, stomach and esophageal carcinoma (STES), STAD, LIHC, PAAD. HAS2 was significantly positively correlated with TMB in four types of tumors, namely GBMLGG, LGG, SARC, THYM; and significantly negatively correlated with TMB in nine types of tumors, namely LUAD, COAD, colon adenocarcinoma/rectum adenocarcinoma esophageal carcinoma (COADREAD), ESCA, PRAD, HNSC, LUSC, LIHC, THCA. HAS3 was significantly positively correlated with TMB in six types of tumors, namely GBMLGG, LGG, Pan-kidney cohort (KIPAN), UCEC, HNSC, THYM; and significantly negatively correlated with TMB in 10 types of tumors, namely LUAD, BRCA, ESCA, STES, STAD, PRAD, LUSC, THCA, SKCM, CHOL. Similarly, HAS1 was observed to be significantly positively correlated with MSI in two types of tumors, namely GBMLGG and THCA; and significantly negatively correlated with MSI in five types of tumors, namely ESCA, STES, KIPAN, STAD, HNSC. HAS2 was significantly positively correlated with MSI in two types of tumors, namely SARC and testicular germ cell tumors (TGCT); and significantly negatively correlated with MSI in 10 types of tumors, namely GBMLGG, LUAD, COAD, COADREAD, STES, KIPAN, PRAD, HNSC, LUSC, DLBC. HAS3 was significantly positively correlated with MSI in three types of tumors, namely UCEC, LUSC, KICH; and significantly negatively correlated with MSI in seven types of tumors, namely GBMLGG, ESCA, STES, KIPAN, STAD, rectum adenocarcinoma (READ), DLBC.

### 3.6 Analysis of DNA methylation and protein phosphorylation, acetylation, ubiquitination in HAS

**Fig. 4:**
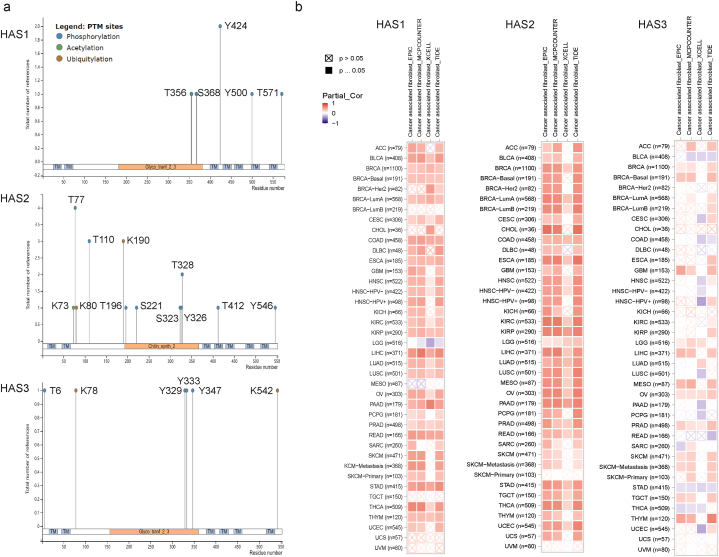
Protein modification and immune infiltration of HAS1, 2, and 3. (a) Phosphorylation, acetylation, and ubiquitination sites have been reported for the three types of HAS. (b) HAS1, 2, and 3 expression and immune infiltration of CAFs.

DNA methyltransferase 1 (DNMT1) is the most common and important methyltransferase in the human body. DNMT1 is a methyltransferase that plays a role in maintaining DNA methylation patterns during cell division, ensuring genome stability and normal gene expression. DNMT1 is mainly located in the nucleus and can recognize hemimethylated CpG sequences, adding back lost methyl groups to ensure that the genome methylation pattern in daughter cells after cell division is the same as that in the mother cell. Upregulation of DNMT1 expression can cause tumor suppressor genes (TSGs) to undergo methylation, thereby inhibiting their expression and promoting tumor development. Table 2 summarizes the correlation between the three HAS genes and DNMT1, showing that all three genes are significantly positively correlated in ACC, LGG (except for the negative correlation of HAS1), and TGCT. Taking LGG as an example, the methylation sites of the three genes in LGG are visualized (Fig. S5). Fig. 4a shows previously reported phosphorylation, acetylation, and ubiquitination sites, which have differences.

**Table 2:** Correlation of HAS1, 2, 3 and DNA methyltransferases 1 (DNMT1) in TCGA Tumor.

Cancer name	Gene A	Gene B	p value	R
ACC	DNMT1	HAS1	0.039	0.24
		HAS2	0.011	0.29
		HAS3	0.012	0.28
BLCA	DNMT1	HAS1	0.12	0.078
		HAS2	0.29	0.052
		HAS3	0.028	−0.11
BRCA	DNMT1	HAS1	0.89	−0.0042
		HAS2	0.37	0.027
		HAS3	0.76	0.0091
CESC	DNMT1	HAS1	0.21	−0.072
		HAS2	0.47	−0.041
		HAS3	0.64	−0.027
CHOL	DNMT1	HAS1	0.92	−0.018
		HAS2	0.042	0.34
		HAS3	0.34	−0.16
COAD	DNMT1	HAS1	0.18	−0.082
		HAS2	0.13	0.091
		HAS3	0.0039	0.17
DLBC	DNMT1	HAS1	0.23	0.18
		HAS2	0.79	0.039
		HAS3	0.027	0.32
ESCA	DNMT1	HAS1	0.52	−0.048
		HAS2	0.58	0.042
		HAS3	0.5	0.051
GBM	DNMT1	HAS1	0.26	−0.088
		HAS2	0.019	0.18
		HAS3	0.32	0.079
HNSC	DNMT1	HAS1	0.99	−0.00048
		HAS2	0.98	−0.0012
		HAS3	0.26	−0.049
KICH	DNMT1	HAS1	0.54	0.077
		HAS2	0.009	0.32
		HAS3	0.053	0.24
KIRC	DNMT1	HAS1	0.74	0.015
		HAS2	1.3e−15	0.34
		HAS3	5.8e−08	0.23
KIRP	DNMT1	HAS1	0.52	0.038
		HAS2	0.00014	0.22
		HAS3	2.7e−09	0.34
LAML	DNMT1	HAS1	0.57	0.043
		HAS2	0.99	0.0014
		HAS3	0.0015	0.24
LGG	DNMT1	HAS1	3.8e−07	−0.22
		HAS2	5.5e−08	0.24
		HAS3	9.6e−56	0.62
LIHC	DNMT1	HAS1	0.004	0.15
		HAS2	0.067	0.096
		HAS3	0.00012	0.2
LUAD	DNMT1	HAS1	0.62	−0.022
		HAS2	0.071	0.082
		HAS3	5.4e−08	0.24
LUSC	DNMT1	HAS1	0.21	0.057
		HAS2	0.071	0.082
		HAS3	0.12	0.07
MESO	DNMT1	HAS1	0.01	−0.27
		HAS2	0.0031	0.31
		HAS3	0.12	0.17
OV	DNMT1	HAS1	0.35	−0.045
		HAS2	0.91	0.0055
		HAS3	0.00019	0.18
PAAD	DNMT1	HAS1	0.0014	0.24
		HAS2	0.0016	0.23
		HAS3	0.2	0.096
PCPG	DNMT1	HAS1	0.45	−0.057
		HAS2	0.81	−0.018
		HAS3	0.072	0.13
PRAD	DNMT1	HAS1	0.2	−0.058
		HAS2	0.0053	0.13
		HAS3	0.032	0.096
READ	DNMT1	HAS1	0.76	−0.033
		HAS2	0.18	0.14
		HAS3	0.00067	0.35
SARC	DNMT1	HAS1	0.4	−0.052
		HAS2	0.01	0.16
		HAS3	3.3e−05	0.25
SKCM	DNMT1	HAS1	0.61	0.024
		HAS2	0.62	0.023
		HAS3	0.35	−0.044
STAD	DNMT1	HAS1	0.39	−0.043
		HAS2	0.52	0.032
		HAS3	0.97	−0.0018
TGCT	DNMT1	HAS1	0.036	0.18
		HAS2	4.5e−07	0.42
		HAS3	7.4e−08	0.44
THCA	DNMT1	HAS1	0.097	0.074
		HAS2	2.9e−11	0.29
		HAS3	0.95	−0.0028
THYM	DNMT1	HAS1	0.074	−0.17
		HAS2	0.23	−0.11
		HAS3	0.57	−0.053
UCEC	DNMT1	HAS1	0.59	−0.041
		HAS2	0.5	0.052
		HAS3	0.78	0.021
UCS	DNMT1	HAS1	0.46	−0.1
		HAS2	0.29	−0.14
		HAS3	0.87	−0.022
UVM	DNMT1	HAS1	0.031	0.24
		HAS2	0.42	−0.092
		HAS3	1.9e−09	0.61

### 3.7 Immune infiltration analysis

Table S1 reflects the correlation between immune cell infiltration (based on seven algorithms, including 21 types of immune cells such as B cells, T cells, macrophages, etc.) and three genes in TCGA cancer patients, with items marked in red for p < 0.05. Cancer-associated fibroblasts (CAFs) can secrete a large amount of ECM molecules, such as collagen, abd HA, which can promote the formation and transformation of the TME, thus providing support for tumor growth and metastasis. HAS1 and HAS2, as key enzymes in HA synthesis, are significantly positively correlated with the degree of CAF infiltration in most cancers, but HAS1 is significantly negatively correlated with the degree of CAF infiltration in LGG. The correlation between HAS3 and CAF was not very significant (Fig. 4b).

### 3.8 Network analysis and expression data reveal interacting proteins and correlated genes in HAS expression

**Fig. 5:**
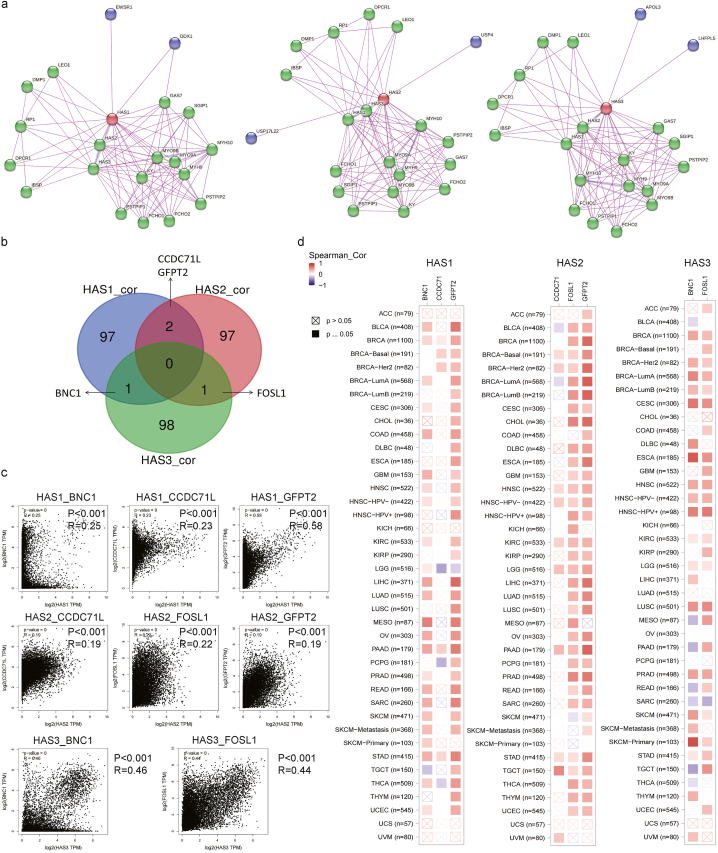
Analysis of proteins related to HAS. (a) Available experimentally determined HAS1, 2, 3-binding proteins. Red circles represent the search terms, green circles represent the same interacting proteins, and purple circles represent the unique interacting proteins for each gene; (b) intersection analysis of the top 100 HAS1, 2, and 3-correlated genes; (c) the scatter plot shows the correlation between intersecting genes and HAS; (d) the heatmap displays in detail the correlation between the intersecting genes and HAS in different types of tumors.

Using the STRING tool, the top 20 interacting proteins were obtained, all of which have experimental evidence supporting their interaction. Fig. 5a shows that the three HAS genes share a majority of common interacting proteins (such as MYH10, KY, RP1, and IBSP) and a few specific interacting proteins (such as DDX1, EWSR1, USP4, USP17L2, APOL3, and LHFPL5). Using the GEPIA2 tool in combination with all tumor expression data from TCGA, the top 100 genes related to the expression of the three HASs were respectively obtained, sorted by Pearson correlation coefficient and summarized in Table S2. The intersection of these 300 genes was taken, revealing CCDC71L and GFPT2 as the intersecting genes between HAS1_cor and HAS2_cor, BNC1 as the intersecting gene between HAS1_cor and HAS3_cor, and FOSL1 as the intersecting gene between HAS2_cor and HAS3_cor (Fig. 5b). Fig. 5c details their R and p values in all TCGA tumor and normal samples. In addition, a heat map of their correlation in specific tumor types was also generated (Fig. 5d).

### 3.9 Enrichment analysis reveals potential pathways and functions associated with interacting proteins and related genes of HAS in cancer

**Fig. 6:**
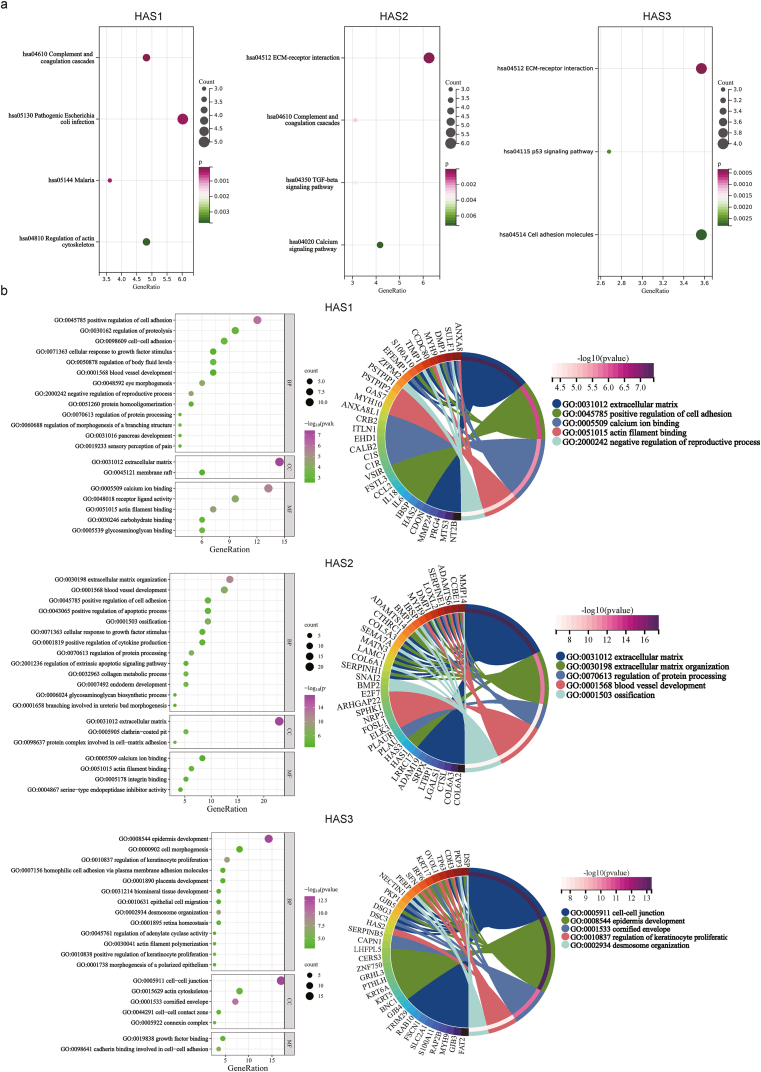
Enrichment analysis of the HAS-associated protein pathways. (a) KEGG; (b) GO.

Next, we merged 20 interacting proteins and 100 related genes for KEGG and GO enrichment analyses. The KEGG bubble plot (Fig. 6a) suggests that “complement and coagulation cascades”, “pathogenic escherichia coli infection”, “mlaria”, and “regulation of actin cytoskeleton” may be involved in the oncogenic effects of HAS1. “ECM-receptor interaction”, “complement and coagulation cascades”, “TGF-beta signaling pathway”, and “calcium signaling pathway” may be involved in the oncogenic effects of HAS2. “ECM-receptor interaction”, “p53 signaling pathway”, and “Cell adhesion molecules” may be involved in the oncogenic effects of HAS3. Fig. 6b shows bubble plots for the respective GOBP, GOCC, and GOMF categories, and the top five enriched functional categories for each gene set are highlighted.

### 3.10 Cell experiments show that HAS2 promotes the proliferation and migration of GBM

**Fig. 7:**
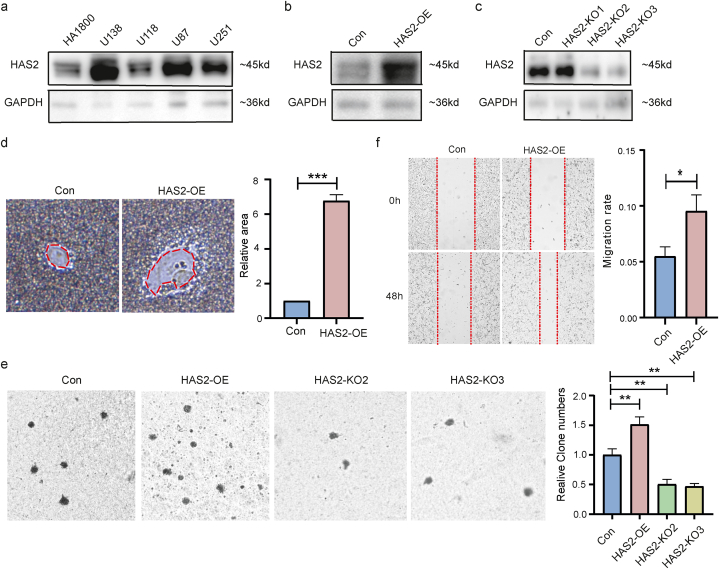
Phenotypic experiments on GBM cells. (a) The Western blot results showed a significant increase in the expression levels of HAS2 in U138, U87 and U251 cells; successfully constructed HAS2-OE (b) and HAS2-KO (c) cells. (d) U138-HAS2-OE cells secrete more HA; (e) HAS2-OE promotes the proliferation of GBM cells, while HAS2-KO has the opposite effect; (f) GBM cells with HAS2-OE have a higher migration rate than the control cells. *P < 0.05, **P < 0.01, ***P < 0.001.

Finally, we preliminarily validated the impact of HAS2 on tumor development in GBM cell lines. The expression level difference of HAS2 between human normal astrocyte cell line (HA1800) and GBM cell lines (U87, U118, U138, and U251) was compared using western-blot method. The results showed that the expression level of HAS2 was significantly increased in U138, U87, and U251 cells (Fig. 7a). We successfully constructed U138-HAS2-OE (Fig. 7b) and U138-HAS2-KO (Fig. 7c) cell lines for subsequent experiments. HAS2-OE promoted U138 cells to secrete more HA (Fig. 7d), and promoted cancer cell proliferation (Fig. 7e) and migration (Fig. 7f).

## 4 Discussion

HA is a type of glycosaminoglycan, a long chain of carbohydrate molecules that are naturally present in the human body. It is a key component of the ECM and is involved in various biological processes such as wound healing, tissue repair, and cell signaling. HA is produced through a series of enzymatic reactions by three enzymes called HAS1, HAS2, and HAS3. These enzymes work in concert to produce HA from precursor molecules, such as uridine diphosphate (UDP)-glucuronic acid and UDP-N-acetylglucosamine. The production of HA is regulated by various factors, including growth factors, cytokines, and mechanical stress. HA plays a critical role in regulating biological processes across different species, where HA molecules of varying sizes, under normal and pathological conditions, exert specific effects on cell signaling, function, and morphology based on their interactions with receptors [5]. To date, there have been no reports of a pan-cancer analysis of HAS, and whether they play a role in different tumors through the same molecular mechanisms remains unexplored. Therefore, we utilized public databases and tools to provide a comprehensive description of the characteristics of HAS1, 2, and 3 across cancers and the potential biological pathways involved at the molecular, protein, cellular, and clinical sample levels.

In this study, we investigated three genes responsible for HA synthesis by analyzing their chromosomal location, structure, and systematic evolutionary tree. Gene localization is crucial in determining whether a gene is linked to a particular disease or biological process. Our analysis showed that HAS2 is located outside the predicted critical region for langer-giedion syndrome, making it an unlikely candidate gene for this genetic syndrome. Furthermore, the protein structures of HAS1, 2, and 3 are conserved across different species, indicating that these proteins have similar mechanisms and physiological functions. Thus, analyzing protein structural similarity can provide insights into their potential physiological functions and mechanisms. Dysregulation of the three genes often occurs in cancer, contributing to tumor progression and metastasis. High expression levels of HAS1 and HAS2 are associated with poor prognosis in various types of cancer, such as ovarian, breast, and pancreatic cancer.

Isoenzymes may play different roles in various cancers, often related to their distinct functions in cell metabolism and signal transduction pathways. Based on the fact that these three genes are members of the HAS family, they share a significant number of common interacting proteins and participate in the same pathways. However, they also have structural differences, resulting in a few specific interacting proteins and pathways. The function of the three genes in normal tissues and tumors exhibits both commonalities and specificities, and in some cancers, even opposite trends. For instance, in LGG, HAS2 expression is upregulated and positively correlated with CAF infiltration, but negatively correlated with infiltration of B cells, CD8^+^ T cells, and myeloid dendritic cells (mDCs), which was already testified by scientists showing that HA impairs the function of immune cells by reducing their migration and activation; therefore, high levels of HAS2 expression are often associated with reduced immune cell infiltration and poor prognosis in various types of cancer, such as breast and pancreatic cancer [23,24]. Therefore, targeting the reduction in HAS2 expression or decreasing the accumulation of HA has been shown to enhance immune cell infiltration and improve anti-tumor immunity in preclinical models. Conversely, our analysis showed that HAS1 has the opposite trend. In addition to their roles in immune cell infiltration, both HAS1 and HAS2 participate in the complement and coagulation cascades, which are crucial for injury repair and hemostasis. However, HAS2 and HAS3 participate in ECM-receptor interaction, and specific endothelial cell ECM receptors lead to changes in vascular morphology during wound repair. Furthermore, HAS2 mainly participates in the TGF-beta signaling pathway, which was consistent with Siriwan Ongchai and H Porsch's report that HAS2 can be activated by TGF-beta, leading to increased hyaluronic acid (HA) synthesis and accumulation, in turn, the accumulation of HA can promote TGF-beta signaling by regulating the availability and activity of TGF-beta in the ECM [25,26]. Our analysis showed that HAS3 participates in the p53 signaling pathway. However, there is limited evidence to suggest that the HAS3 gene is directly involved in the p53 pathway, and further research is needed to fully understand the nature of this relationship.

In addition, our analysis showed that the HAS1, 2, and 3 genes are crucial for wound healing and angiogenesis. To support the analysis, our particle exclusion experiment shows that the overexpression of HAS2 promotes the secretion of HA in GBM. GBM is the most aggressive and common form of malignant brain tumors with distinct molecular characteristics and complexities that set them apart from other cancers. Understanding the specific genetic alterations, that drive GBM development and progression is essential for developing targeted therapies tailored to its unique biology. HAS2 has been implicated in promoting tumor aggressiveness and the formation of the extracellular matrix, which contributes to GBM growth and invasion. Investigating the role of HAS2 in GBM can provide valuable insights into its potential as a therapeutic target. Soft agar experiments show that HAS2 promotes the proliferation of GBM cells. This is consistent with the findings of Yoo et al. [27] and others, who reported that the recurrence of GBM after radiation therapy is attributed to the increase of HAS2 induced by radiation, which increases the production of HA and subsequently activates carcinogenic signaling pathways. Wound healing experiments showed that overexpression of HAS2 significantly enhanced cell migration ability.

The limitations of our study are that most of our data relied on public databases, which may differ in gene sequencing technology, sample types, data quality, and data processing. Additionally, gene expression may vary among different subtypes of some cancers, which could affect the accuracy of pan-cancer analysis. Furthermore, pan-cancer analysis often requires the annotation of gene function to identify which pathways and biological processes may have an impact on cancer occurrence and development. However, our understanding of the function of some genes is still limited, and there may be uncertainties in the annotation of gene function. Finally, we only conducted proliferation and wound healing experiments in GBM cells that were already available in the laboratory. In future studies, more cancers and methods should be supplemented to explore the molecular mechanisms.

## 5 Conclusion

Our study indicates that HAS1, 2, and 3 share commonalities and unique characteristics across different types of cancer. They have varying roles in carcinogenesis and prognosis and hold potential as clinical targets for cancer treatment.

## Author contribution statement

Xunxia Bao: Conceived and designed the experiments; Performed the experiments; Analyzed and interpreted the data; Contributed reagents, materials, analysis tools or data; Wrote the paper.

Juan Ran: Analyzed and interpreted the data; Contributed reagents, materials, analysis tools or data; Wrote the paper.

Chuifang Kong: Analyzed and interpreted the data; Wrote the paper.

Zunxi Wan; Tengfei Yu; Shengming Ruan; Wenjing Ding: Contributed reagents, materials, analysis tools or data; Wrote the paper.

Juling Wang: Performed the experiments; Analyzed and interpreted the data; Contributed reagents, materials, analysis tools or data; Wrote the paper.

Leiming Xia; Daoxiang Zhang: Conceived and designed the experiments; Analyzed and interpreted the data; Wrote the paper.

## Data availability statement

Data will be made available on request.

Supplementary data to this article can be found online at https://doi.org/10.1016/j.heliyon.2023.e19112.

## Ethics statement

Not applicable. This study did not involve human participants or animals.

## Consent for publication

Not applicable.

## Funding statement

This study was supported by grants from the National Science Foundation of China (82073372).

## Declaration of competing interest

The authors declare that they have no known competing financial interests or personal relationships that could have appeared to influence the work reported in this paper.

## Figures and Tables

**figs1:**
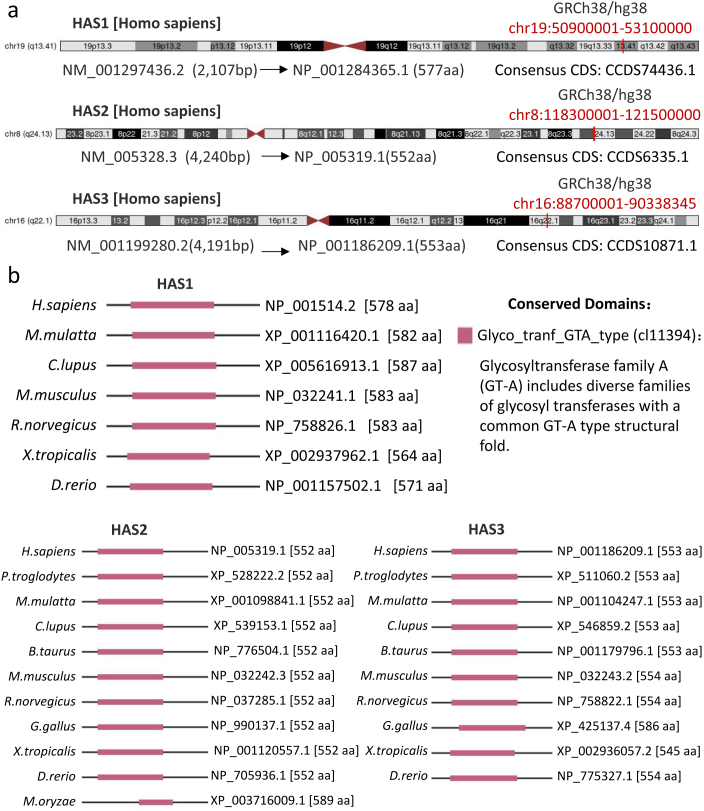


**figs2:**
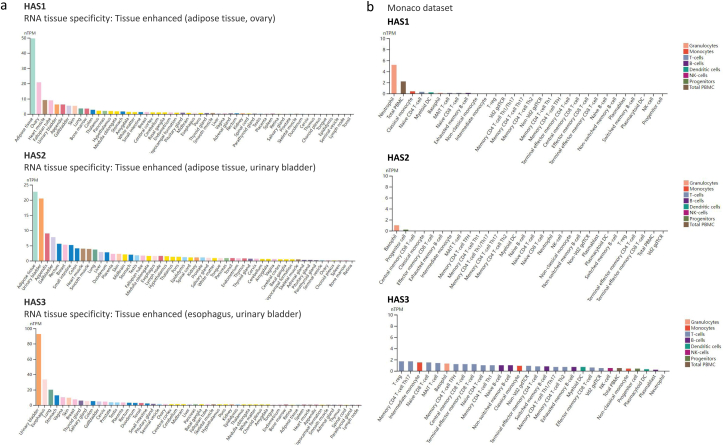


**figs3:**