# Supplementary figures and images for: Pan-cancer analysis reveals the potential of hyaluronate synthase as therapeutic targets in human tumors
Source: Heliyon. 2023 Aug 12;9(8):e19112. doi: 10.1016/j.heliyon.2023.e19112 (PMC10448108; doi:10.1016/j.heliyon.2023.e19112)

HAS1

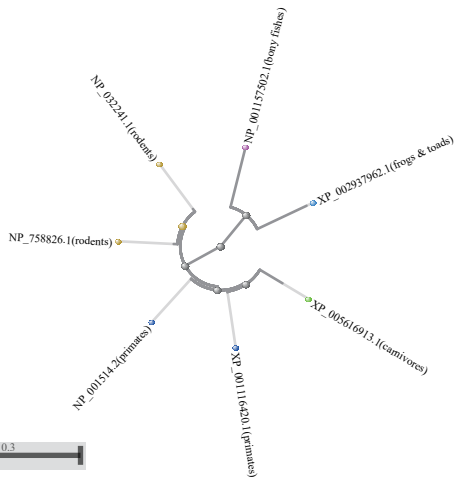

HAS2

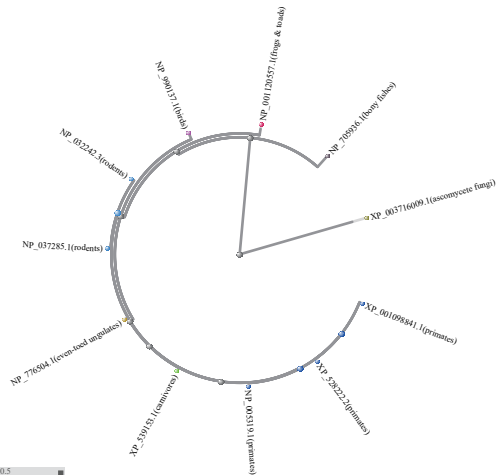

HAS3

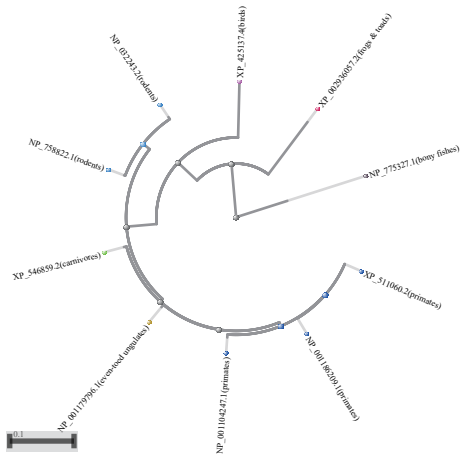

Supplement: Multimedia component 3 [file mmc3.pdf]

**a** HAS1

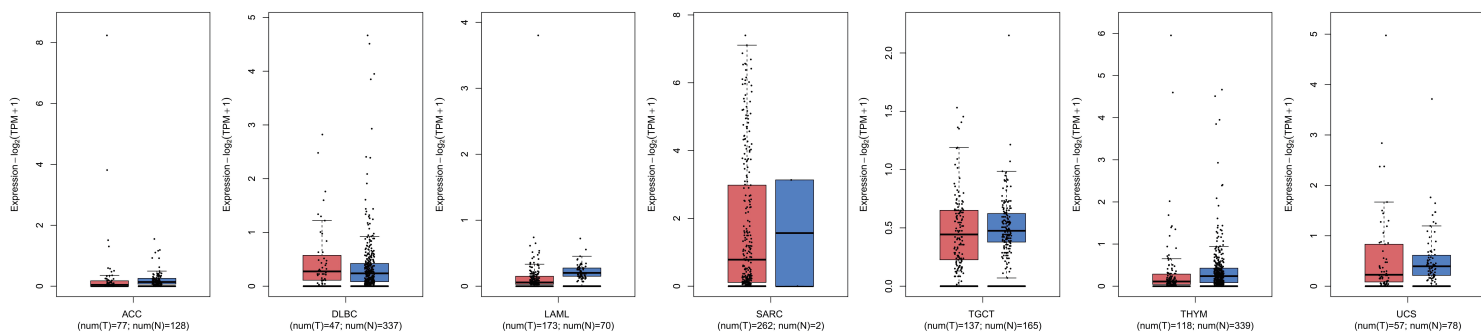

**b** HAS2

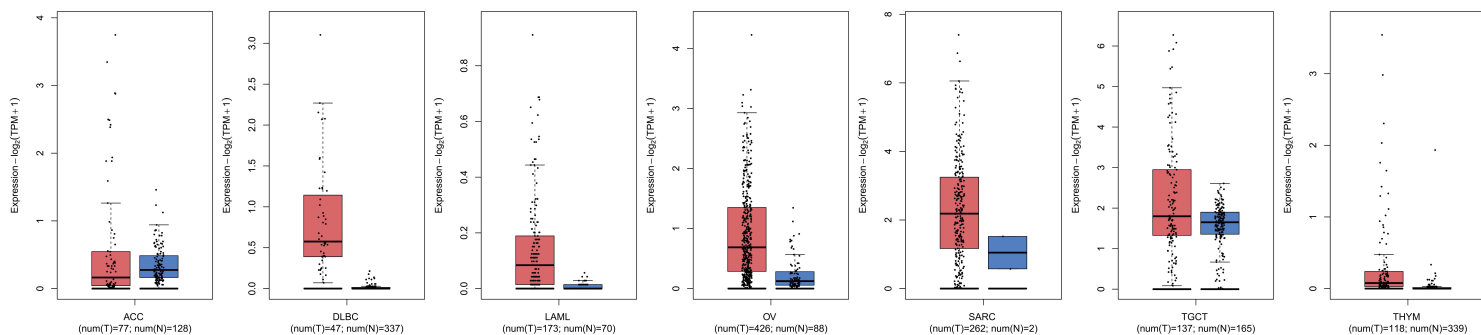

**c** HAS3

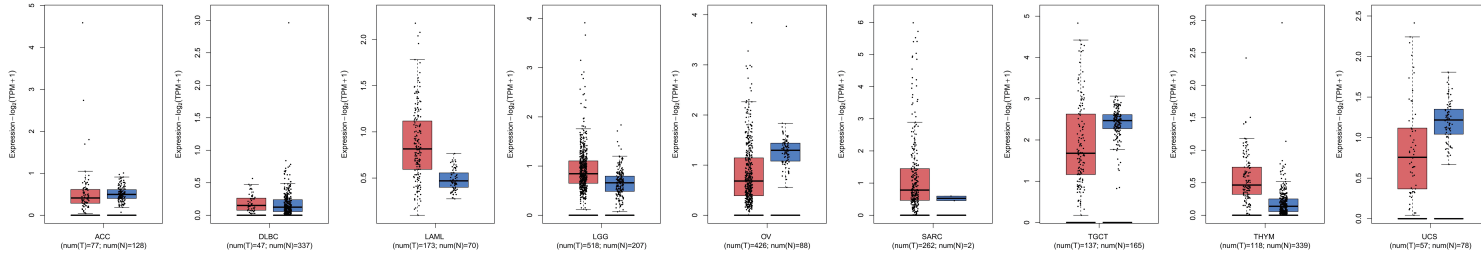

**d** HAS1

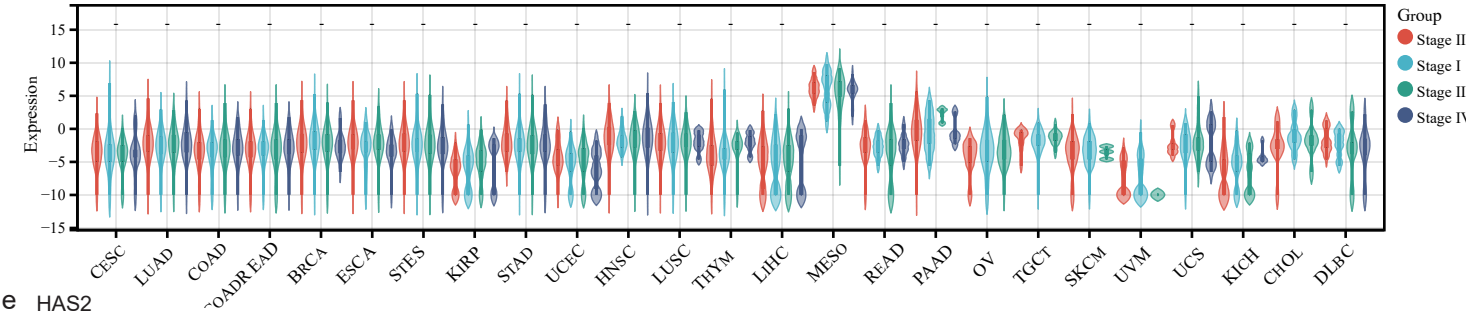

**e** HAS2

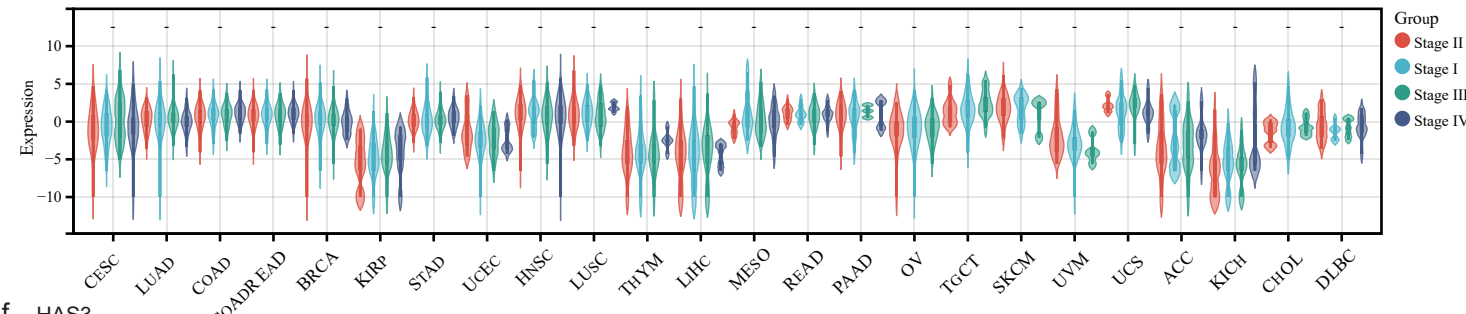

**f** HAS3

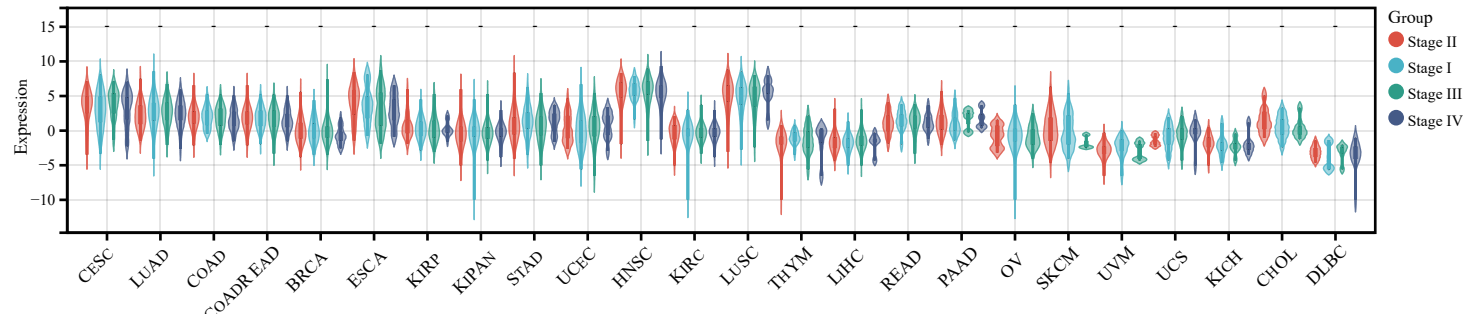

Supplement: Multimedia component 4 [file mmc4.pdf]
