# Supplementary material for: Pan-cancer analysis reveals the potential of hyaluronate synthase as therapeutic targets in human tumors
Source: Heliyon. 2023 Aug 12;9(8):e19112. doi: 10.1016/j.heliyon.2023.e19112 (PMC10448108; doi:10.1016/j.heliyon.2023.e19112)
Supplement: Multimedia component 5 [file mmc5.pdf]

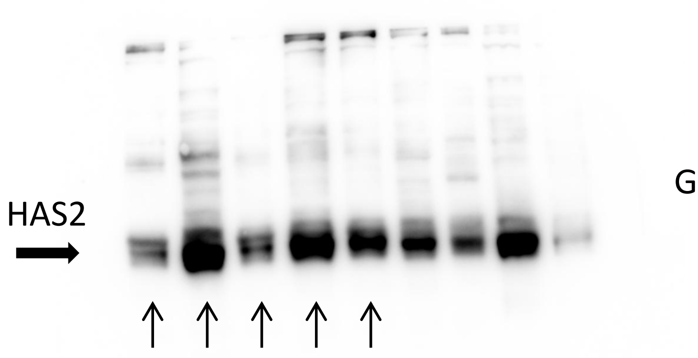

Fig 7a HAS2
(HA1800/U138/U118/U87/U251)

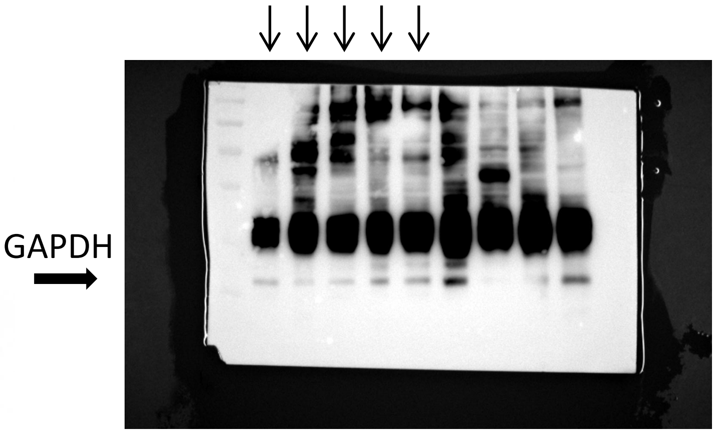

Fig 7a GAPDH
(HA1800/U138/U118/U87/U251)

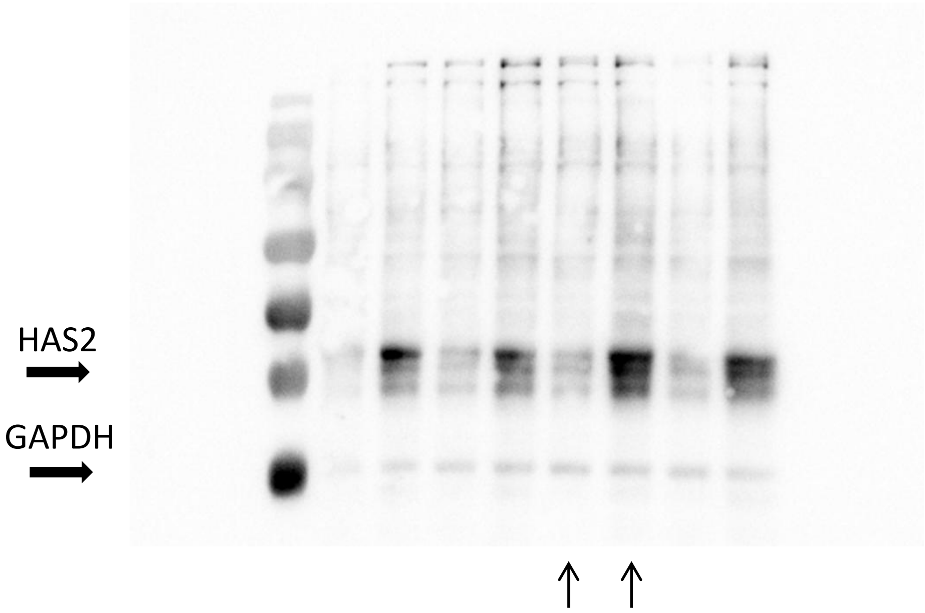

Fig 7b HAS2 GAPDH
(Con/HAS2-OE)

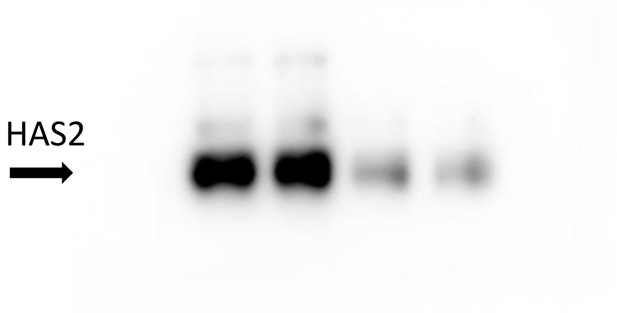

Fig 7c HAS2
(Con/HAS2-KO1/HAS2-KO2/HAS2-KO3)

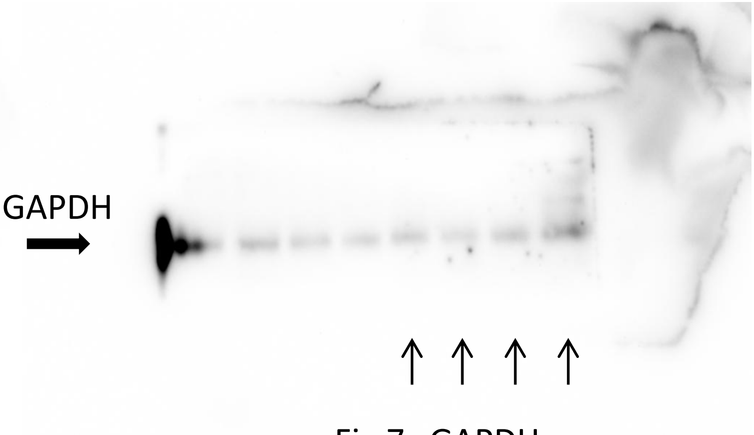

Fig 7c GAPDH
(Con/HAS2-KO1/HAS2-KO2/HAS2-KO3)
